# Attack Repertoires in Outbred Male CD1-Mice Are Associated with Nucleus Accumbens Neuroligin-2

**DOI:** 10.1523/ENEURO.0471-25.2026

**Published:** 2026-05-13

**Authors:** Nastacia L. Goodwin, Pranav Anumolu, Natalie P. Hoffman, Ainsley C. Barrow, Valerie S. Tsai, Lukas K. MacMillen, Sam A. Golden, Mitra Heshmati

**Affiliations:** ^1^Center for Excellence in the Neurobiology of Addiction, Pain and Emotion, University of Washington, Seattle, Washington; ^2^Department of Neurobiology and Biophysics, University of Washington, Seattle, Washington; ^3^Undergraduate Program in Neuroscience, University of Washington, Seattle, Washington; ^4^Department of Anesthesiology and Pain Medicine, University of Washington, Seattle, Washington

**Keywords:** aggression, appetitive, CD-1 mice, neuroligin-2, operant, reactive

## Abstract

Aggression may be behaviorally distinguished by reactive or appetitive properties. Here, we use a model of operant aggression administration, in which outbred male CD-1 mice lever press (contingent) or do not lever press (noncontingent) to attack an intruder mouse, to examine behavioral differences in aggression reinforcement. Contingent reinforcement identifies the behavioral and neural basis of appetitive, or rewarding, aggression self-administration, while noncontingent reinforcement isolates reactive, or involuntary, components. Females are not used in this study due to their low propensity to attack. We applied supervised machine-guided behavioral classification and Shapley additive scores (SHAP) to describe differences and similarities in attack behavior features. We find that behavioral sequences of an attack bout are similar whether aggression reinforcement is contingent or noncontingent, though underlying neural mechanisms differ. Fos immunolabeling following operant reinforcement reveals distinct network activity patterns between contingent and noncontingent groups, supporting distinct neural mechanisms in appetitive or reactive aggression. We further identify high Fos activity in nucleus accumbens (NAc) in both contingent and noncontingent groups. NAc activity and changes in NAc neuroligin-2 (NLGN2) expression are associated with aggressive behavior. We find that the number of attack bouts is negatively correlated with NAc NLGN2 immunolabeling, regardless of contingent or noncontingent intruder presentation. As a result, molecularly dissociable features do not necessarily reflect operant behavioral repertoires.

## Significance Statement

Aggressive behavior presents for different reasons, and two **important** clinical presentations include appetitive (sought after) and reactive (fight or flight) aggression. Understanding how these phenotypes differ in their behavioral and neural signatures is essential for identifying mechanisms at the intersection of neuropsychiatric disease and maladaptive aggression. Using an operant model in outbred male CD-1 mice, we combined explainable machine learning and forebrain Fos mapping to compare contingent (appetitive) and noncontingent (reactive) aggression. We find that, although behavioral repertoires are notably similar across contingency contexts, neural activity and nucleus accumbens neuroligin-2 expression distinguish aggression severity and phenotype. These findings reveal that molecular features can diverge from behavior, providing new insight into the neurobiology of contingent and noncontingent aggression.

## Introduction

Appetitive (rewarding) aggression engages neural mechanisms that are distinct from reactive (defensive) aggression (Lin et al., 2011; Anderson, 2012; Falkner et al., 2016; Golden et al., 2016, 2017). The cellular and synaptic mechanisms that distinguish appetitive from reactive aggression are still unclear and an area of active study. The operant aggression self-administration procedure, in which aggressive male mice learn to lever press for access to fight a subordinate intruder mouse, provides a platform for interrogating mechanisms underlying the motivation to attack (Golden et al., 2017). Noncontingent administration interrogates the mechanisms underlying reactive aggression while maintaining similar frequency and duration of forced social interactions within the same environmental context as contingent self-administration. Females are not used in this study due to their low propensity for attack behavior following contingent social partner administration (Aubry et al., 2022). We hypothesized that the individual behavioral attack repertoires selected by aggressive mice during contingent and noncontingent aggression are unique and can be behaviorally distinguished depending on underlying motivation. To distinguish these differences, we use supervised machine learning analysis of attack bouts following intruder presentation (Goodwin et al., 2020, 2024) with an emphasis on explainability metrics to reveal the relevant biological features (Goodwin et al., 2022). To examine contingent and noncontingent motifs in parallel, we adapted the operant aggression self-administration procedure to include a group of mice that receive noncontingent intruder presentations that are not linked to lever pressing (pseudo-yoked control), alongside mice that receive contingent intruder presentations paired to lever presses. We then analyze the attack repertoires engaged during the contingent (appetitive) or noncontingent (reactive) aggressive interactions within the same operant context.

Using supervised approaches paired with explainability metrics, we previously showed that simply changing the size of an arena can significantly influence the attack repertoires used by mice during reactive noncontingent aggression (Goodwin et al., 2024). As a result, here we model reactive, noncontingent aggression within the same operant context. Explainability helps communicate and compare classifier behavior and supports informed model selection and use in behavioral neuroscience (Ribeiro et al., 2016; Sundararajan et al., 2017; Hatwell et al., 2020; Lundberg et al., 2020). We complement our supervised analyses with SHAP (Shapley Additive exPlanations; Lundberg and Lee, 2017), an open-source, well-documented, and actively maintained framework that assigns each feature a value representing its contribution to the difference between a model's baseline (expected) output and actual prediction. This allows us to rank and visualize the features most influential in bouts occurring during contingent or noncontingent contexts. This computational approach is well suited to address if similar behavioral differences exist during contingent and noncontingent aggression.

Bridging behavioral analysis between operant metrics and supervised analysis of freely moving behavior allows for a comprehensive analysis of differences and similarities across contingent and noncontingent aggression reinforcement. We use this framework to compare the resulting attack repertoires and mapped neural activity with Fos immunolabeling across forebrain regions, including nucleus accumbens (NAc), and quantified NAc expression of the inhibitory postsynaptic protein neuroligin-2 (NLGN2). NAc principal medium spiny neurons play a key role in regulating motivated behavior, and we previously identified that chemogenetic activation of NAc modulates aggression seeking using operant procedures (Golden et al., 2019). NAc is also enriched in NLGN2, a postsynaptic adhesion protein that forms the inhibitory GABAergic synapse. Neuroligin gene mutations are associated with autism spectrum disorder and schizophrenia, neuropsychiatric disorders that may present with aggression as a possible disease manifestation. In studies using transgenic mice, we found that cell-type–specific knockdown of NLGN2 in NAc principal neurons promotes dominance and reactive aggressive behavior. We did not previously evaluate the role of NAc NLGN2 in appetitive aggression.

In the following experiments, we test the hypothesis that contingent and noncontingent aggression are characterized by distinct behavioral attack repertoires, forebrain Fos activity patterns, and NAc NLGN2 expression using groups of mice experiencing either contingent or noncontingent aggression self-administration within the same operant procedure. We find that contingent and noncontingent aggression phenotypes are indistinguishable by common metrics such as number of attack bouts but have different underlying structures as evidenced by significantly different SHAP scores. Despite commonality in behavior metrics such as attack bouts, aggression phenotypes are distinguishable in their forebrain patterns of Fos neural activation. Last, we also find that NLGN2 is strongly correlated with attack bouts and aggression level, regardless of contingent and noncontingent reinforcement. Together, these data indicate that molecularly dissociable features may be reflected by grossly similar attack behaviors, independent of operant context.

## Materials and Methods

### Animals

Residents were white coat-colored >4-month-old retired CD1 male breeder mice (CRL: 022). Mice were shipped from Charles River Laboratories with dividers and subsequently housed independently throughout testing. Intruders were 6–8-week-old group–housed c57 male mice bred in-house. We used black coat-colored c57 intruders (Jax, C57BL/6J Strain 000664) against all white coat-colored residents. All mice were housed with *ad libitum* access to food and water on a reverse 12:12 light cycle. All resident mice were single-housed and given enrichment (cotton padding only). Intruder mice were group-housed (up to five per cage) with the same enrichment. Females are not used in this study due to their low propensity for attack behavior following contingent partner administration (Aubry et al., 2022). This study was carried out in accordance with the recommendations in the Guide for the Care and Use of Laboratory Animals published by the National Research Council. The protocol was approved by the University of Washington Institutional Animal Care and Use Committee.

### Experiments

#### Experiment 1: operant aggression self-administration

##### Resident intruder screening

Resident CD1 mice were tested in their home cage with white nesting material temporarily removed for visibility. Same-sex c57 intruder mice were placed into the center of the home cage, and animals were allowed 10 min to freely interact. Animals were monitored for intensity of fighting and any injuries, and assays were immediately stopped at any sign of wounding. Animals underwent 3 consecutive days of 10 min testing, during which they were scored as not aggressive (zero bouts) or aggression (one or more bouts).

##### Aggression self-administration

Mice were trained in a specialized operant box with a guillotine door and side cannister for intruder mice as described earlier (Golden et al., 2017). For contingent self-administration, mice were able to press for access to an intruder on an FR1 schedule for 12 2 min trials, with 2 min intertrial intervals per day. Animals had access to an empty food port with beam break readings and an inactive lever at all times. During trials, a house light went on in the chamber, followed 10 s later by an active lever extending. Following lever press, a 2 s tone sounded, and the guillotine door opened 5 s after lever press. An intruder mouse was ushered into the chamber with the resident, and the guillotine door closed 17 s after lever press. For noncontingent self-administration, average presses per day were calculated across a prior group of self-administration animals under identical testing parameters, and intruders were presented to noncontingent mice at this frequency per day.

##### Pose estimation

All video-recorded behaviors were filmed from above at 30FPS by Basler or WhiteMatter cameras at resolutions ranging from 320 × 300 to 1,550 × 1,050. Performance was assessed on videos of varying resolution. Videos were cropped and trimmed into clips where two mice were present, using the SimBA video preprocessing module, prior to pose estimation using DeepLabCut (Version 1.0-2.2). In DeepLabCut, we tracked seven points per animal (nose, ears, sides, body center, tail base) per animal and labeled frames from a diverse set of videos to create a training set for a ResNet 50 based neural network which tracked both the white test mouse as “Mouse 1” and the c57 intruder as “Mouse 2.” Pose estimation was loaded into SimBA, followed by outlier correction (location criteria, 2.0; movement criteria, 1.0) and video FPS and pixel/millimeter calibration.

##### Behavioral classifiers

We used previously created behavioral classifiers [detailed in Goodwin et al. (2024)] to classify the probability of a behavior of interest happening in each individual video frame. Briefly, videos from diverse lighting and testing conditions were hand scored for behaviors of interest by a trained observer, and these annotations were loaded with pose data into SimBA. Annotations and pose were then used to calculate hundreds of features between animals (nose to nose distance, distance moved over last 50 ms, etc.), and these feature values were used to train random forest classifiers to detect behavior. Hyperparameters for our provided classifiers consist of the following: criterion, entropy; max features, square root; minimum sample leaf, 1; and number of estimators, 2,000. Once probabilities per frame were detected, we set probability threshold cutoffs for positive frames at 0.5 for attack, 0.65 for escape, and 0.45 for defensive, in the resident intruder videos. Thresholds for the operant videos were attack 0.5, escape, 0.65, and defensive 0.45. Thresholds vary across settings due to changes in lighting and resolution, but classifier performance is similar across tests. To account for flickering in behavioral detection and to better segment behavior into meaningful bouts, we applied a Kleinberg filter with the hierarchical search function for all three classifiers to smooth bouts (sigma, 0.03; kappa, 2; hierarchy, 2 for attack, 3 for defensive and escape).

During hand annotation, each behavior had a strict operational definition, as follows:

Attack—Clear physical antagonistic interaction initiated by the resident, characterized by tussling, biting, boxing, and/or corralling.

Defensive—Intruder mouse is fighting back against resident by pushing or biting. Intruder is not instigating aggression.

Escape—Intruder mouse is running away or attempting to run away from resident.

Sniffing—Resident nose can be seen huffing while it is touching or very close to intruder face, body, or anogenital region.

##### Attack location detection for sniffing and attack

We had previously made classifiers for anogenital, body, and face sniffing in our mice. To identify attack locations, we ran all videos with attack classifiers as well as sniffing classifiers. Each frame of attack was categorized as anogenital, body, or face based on the highest probability sniffing classifier using custom python scripts. Data are shown as the average bouts or duration across all trials per day per animal.

Intruder behaviors were calculated based on the number of escape or defensive behaviors experienced by each resident, rather than by individual intruder.

##### SHAP scores

We calculated SHAP scores as in Goodwin et al. (2024). We used SHAP to evaluate how feature values impact classification probabilities. Owing to the Shapley additivity axiom, we can collapse Shapley value contributions of features that measure similar, general, and often colinear characteristics of the behavior of interest into interpretable physical categories while maintaining consistency, accuracy, and biological relevance. Hence, to aid interpretability, we collapsed the features into seven behaviorally defined feature categories that measure general characteristics of social interactions (i.e., animal distances, resident and intruder movement, intruder movement, resident movement, resident shape, intruder shape, and resident and intruder shape). Each of the seven feature categories contained six temporal windows that represent different frame sampling frequencies (single frame to 500 ms). We calculated SHAP scores on 1,000 positive and negative frames per classifier, using the SimBA SHAP function.

#### Experiment 2: resident–intruder testing

Resident CD1 mice were tested in their home cage. The screening process involved a same-sex intruder C57 mice being placed in the resident home cage, and animals were given 10 min to interact. All nesting material was removed for increased visibility. During these 10 min, the resident mice attack duration toward the intruder C57 was measured and recorded. Assays were terminated immediately at any sign of wounding. The interactions were filmed from above. Each 10 min trial occurred once per day, for 3 consecutive days. At 70–90 min following the final trial, the mice were anesthetized and perfused. Control mice were singly housed behaviorally naive CD-1 animals in their home cage. The brain tissue was sliced and immunolabeled for NLGN2 or Fos using the below immunohistochemistry protocol, in adjacent slices.

#### Immunohistochemistry

##### Coronal IHC brain collection and processing

At 70–90 min following the start of behavioral testing, animals were perfused using 1× PBS followed by formalin. Brains were kept in formalin overnight prior to transfer to 30% sucrose in PBS for 2 d at 4°C. Brains were embedded in OCT and frozen at −80°C. Brains were sliced on a cryostat at 40 μm into four series prior to staining and stored in PBS with 0.01% sodium azide.

For Fos IHC, brain slices were washed for 5 × 10 min in PBS, followed by permeabilization using a solution of 1× PBS + 0.25% Triton X-100 (PBST) for 30 min. Brains were blocked for 1 h in PBST + 5% normal donkey serum (NDS). Primary antibody incubation was in PBST + 5% NDS for 19 h using Synaptic Systems c-*fos* rabbit polyclonal antibody at 1:1,000 dilution (Product ID 226 003, lots 9-89 and 9-92). Slices were then washed five times for 5 min in PBS, followed by a 1 h incubation in 1:500 secondary Alexa Fluor 647 AffiniPure Donkey Anti-Rabbit IgG (H + L; Jackson ImmunoResearch Laboratories, 711-605-152) with 1 μg/ml DAPI. Well plates were covered in tinfoil from the time that secondary antibodies were introduced. Slices were then washed five times for 5 min in PBS and mounted on SuperFrost Plus slides using hard-set VECTASHIELD mounting media. All steps took place at room temperature with gentle shaking, and NDS was freshly reconstituted for each round. Staining was conducted in three different batches with groups equally represented in each batch.

A separate series of slices from the same tissue sets (Exp. 1 Operant Aggression and Exp. 2 resident–intruder testing) was immunolabeled for NLGN-2 with rabbit anti-NLGN2 (1:500, rabbit, ab36602; polyclonal antibody; RRID: AB_881542) followed by secondary antibody staining with donkey anti-rabbit Alexa Fluor 488 (1:500; Jackson ImmunoResearch Laboratories, 711-545-152; RRID: AB_157377) to analyze NAc expression of NLGN-2. The NLGN2 antibody shows high specificity via negative controls and immunoprecipitation and was nonreactive with neuroligin 1 or 3 transfection per the supplier website.

Slides were allowed to dry overnight prior to imaging at 20× on a Keyence microscope. Images were focused on DAPI on a single plane and whole slices were stitched using proprietary Keyence software.

##### Cell counting and analysis

Stitched whole-slice images were converted to ome.tiff format using in-house scripts and calibrated to 0.75488 μm/pixel prior to uploading into the open-source program QuPath (Bankhead et al., 2017).

Through QuPath, brain slices were aligned to the Allen Brain Atlas CCF v.3 using the open-source program Aligning Big Brains and Atlases (ABBA). Alignments were loaded back into QuPath from ABBA, at which point we could calculate Fos-positive neurons per region using a random forest machine learning algorithm (*R*^2^ = 0.99, four slices, one hand counted region each) and normalize by area. NLGN-2 expression within the NAc shell and core subregions was quantified using mean fluorescence measures in the ImageJ software.

##### Figures

Figures are made with biorender.com.

##### Code availability

All code is publicly available on the Golden Lab Github https://github.com/sgoldenlab.

##### Statistics

We performed one- or two-way ANOVAS, without FDR correction, independent *t* tests, or linear regressions using Prism 10.4. We used the SMARTTR (Jin et al., 2025) pipeline for analysis of Fos correlation heatmaps shown in Figure 2.

## Results

### Operant aggression tasks reveal consistent attack behavior

Attack behavior is highly stereotyped and can be characterized by a repertoire of species-specific actions displayed during close interactions with an opponent, as studied in resident–intruder tests (Koolhaas et al., 2013). To assess the relative stereotypy of contingent aggression, we compared aggression motifs in noncontingent versus contingent contexts using an operant aggression self-administration procedure. We first selected for aggressive CD1 mice via assessment across 3 d of resident intruder testing in the home cage. We then randomly divided aggressive animals into contingent or noncontingent groups.

**Figure 1.**
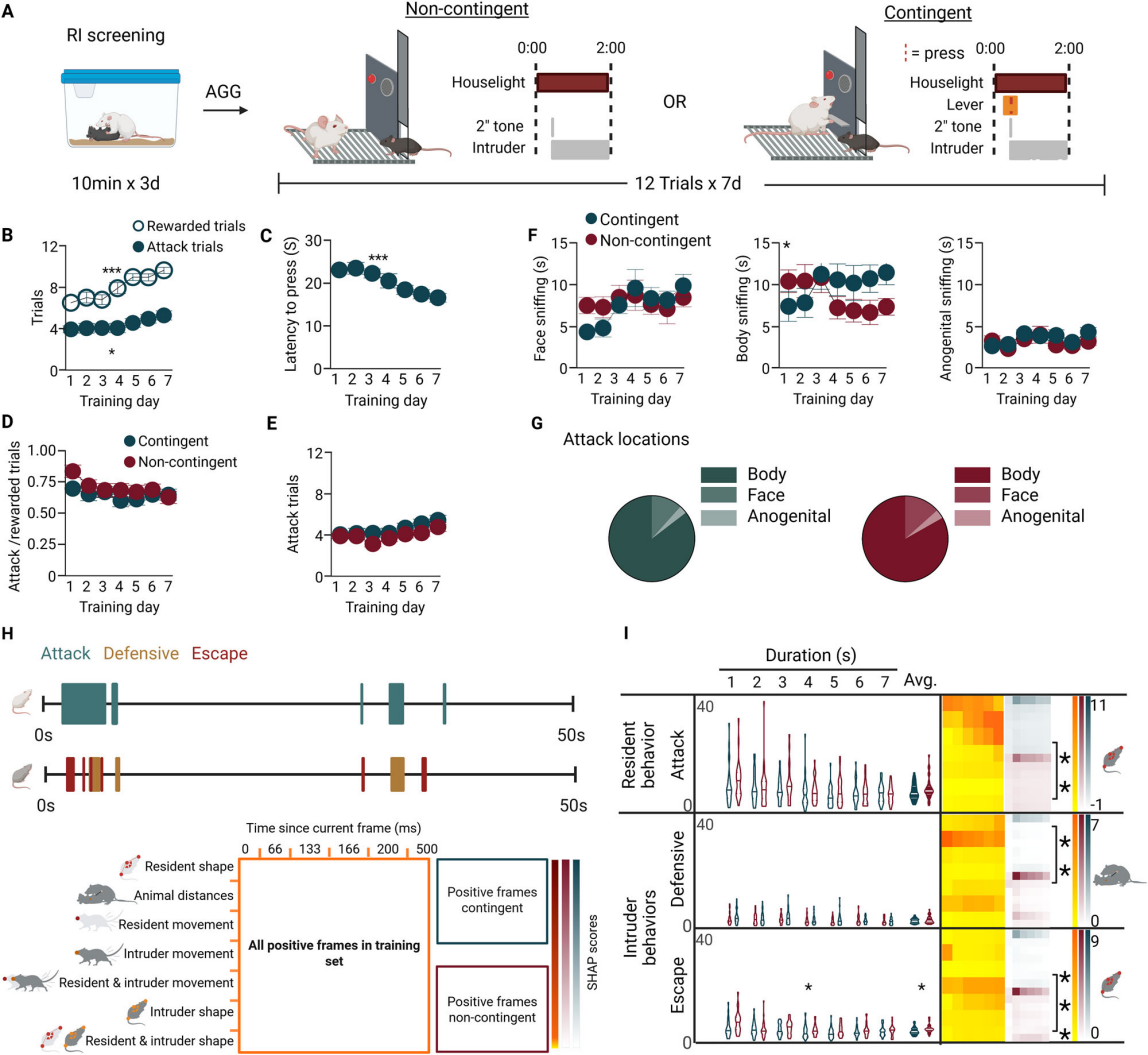
Attack patterns are consistent regardless of contingency in an operant aggression self-administration procedure. ***A***, Experimental schematic. Male CD-1 mice are screened for aggression phenotype and then aggressive mice are trained for social self-administration with either noncontingent (*n* = 31) or contingent (*n* = 35) intruder presentations. ***B***, The number of rewarded and attack trials across days for contingent self-administration. ***C***, Latency to lever press across days for contingent self-administration. ***D***, Ratio of attacks to rewards for noncontingent and contingent mice. ***E***, The number of attack trials across days. ***F***, Time spent sniffing the intruder's face, body, or anogenital areas across days. ***G***, Attack bite locations in contingent (left) versus noncontingent (right) groups. ***H***, Social interactions during rewarded trials were analyzed for attack (resident mice) or escape and defensive coping behaviors (intruder mice) using SimBA supervised machine learning based behavioral classification. A representative Gantt plot of CD1 attack behavior and C57 intruder defensive and escape behavior over a trial is shown. SHAP analysis was performed on all rewarded trials comparing contingent and noncontingent aggressors. An analytical schematic of SHAP feature comparisons shows the biological bins (*y* axis) and time bins (*x* axis) across all mice (large box), contingent mice only (top right box), and noncontingent mice (bottom right box). This framework is used in panel ***I***. ***I***, Left, Supervised behavioral classification for attack (resident), defensive, and escape (intruder) classifiers. Noncontingent mice data are represented in teal, and contingent data are maroon. Total duration (s) across individual testing days (*n* = 31 noncontingent; *n* = 35 contingent). Right, Corresponding SHAP analysis. The biological bin with greatest significance difference between contingent and noncontingent mice is marked with brackets and * in the figure, and the icon to the right indicates what biological bin it is (see schematic). The color intensity for all three SHAP datasets per classifier are on the same scale, indicated by the scales on the right. Asterisks denote significance levels: *p < 0.05, **p < 0.01, and ***p < 0.001. See Table 1 for full statistical analysis.

We define contingent aggression as the acquisition of voluntary lever pressing by aggressive CD-1 outbred male mice to engage in interactions with a young subordinate male c57bl6/j intruder mouse. We define noncontingent aggression as the pseudorandomized, frequency-matched entrance of an intruder mouse leading to interactions between CD-1 outbred male mice and a younger c57bl6/j mouse (Fig. 1*A*). Contingent mice showed increased lever presses and decreased latency to lever press across days (*p* < 0.0001; *F*_(6, 238)_ = 8.871; *p* < 0.0001; *F*_(4.429, 150.6)_ = 7.508, respectively; *n* = 35), indicating they acquired aggression seeking behavior (Fig. 1*B*,*C*). As contingent mice acquired self-administration, they also increased the number of attack bouts over the 7 d trial (*p* = 0.0004; *F*_(4.233, 270.9)_ = 5.119; *n* = 31; Fig. 1*B*). Both noncontingently and contingently reinforced CD-1 mice attacked on a similar number and proportion of trials (*p* = 0.9399; *F*_(6, 384)_ = 0.9399; *n* = 35 contingent, 31 noncontingent; Fig. 1*D*,*E*). Contingent animals showed significantly more body directed sniffing than did noncontingent animals (Fig. 1*F*; *p* = 0.0142; *F*_(7, 222)_ = 2.578). To address the possibility that contingency led to differences in attack severity, commonly measured by assessing attack location (head/face, forequarters, hindquarters), we used custom Python scripts with the SimBA attack classifier data to assess the average seconds of attack per face, body, or anogenital region averaged across trials per day, observing no differences in attack locations by contingency (Fig. 1*F*,*G*). Using supervised behavioral classification (via SimBA), we evaluated global attack characteristics in aggression bouts during contingent and noncontingent self-administration (Goodwin et al., 2024; Fig. 1*H*). We calculated the total duration for attack (CD-1 residents) and escape and defensive behaviors (c57 intruders), both per day of testing and averaged across all seven testing days. We observed no significant differences in resident CD-1 attack duration (Fig. 1*I*, top left). Conversely, while c57 intruders showed no differences in defensive coping behavior, they did escalate escape coping behaviors in response to aggressive bouts during noncontingent self-administration compared with contingent self-administration (*p* = 0.0320; *F*_(6, 351)_ = 2.332; Fig. 1*I*, bottom left). To further analyze the attack features driving supervised classification of aggression between noncontingent and contingent conditions using the same attack classifier, we used explainability metrics (SHAP values) to compare groups. For the attack classifier, we observed that four feature classes showed significant differences: resident movement, resident and intruder movement, intruder shape, and resident and intruder shape (resident movement, *p* = 0.0001; *F*_(5, 5)_ = 71.67 time; *p* = 0.0281; *F*_(1, 5)_ = 9.371 contingency; resident and intruder movement, *p* < 0.0001; *F*_(5, 5)_ = 152.6 time; *p* = 0.0340; *F*_(1, 5)_ = 8.373 contingency; intruder shape, *p* < 0.0001; *F*_(5, 5)_ = 157.2 time; *p* = 0.0010; *F*_(1, 5)_ = 47.40 contingency; resident and intruder shape, <0.0001; *F*_(5, 5)_ = 131.2 time; *p* = 0.0216; *F*_(1, 5)_ = 10.84 contingency). Full statistics for SimBA and SHAP analysis are shown in Table 1.

**Table 1.** Contingent versus noncontingent statistical comparisons for data shown in Figure 1

Figure number	Test	Factor name	*F* value/df	*p* value
Figure 1*B*. Rewarded trials	One-way ANOVA	Day	*F*_(6, 238)_ = 8.871	<0.0001
Figure 1*B*. Attack trials	One-way ANOVA	Day	*F*_(6, 238)_ = 1.625	0.1408
Figure 1*C*. Latency to press	One-way ANOVA	Day	*F*_(4.429, 150.6)_ = 7.508	<0.0001
Figure 1*D*. Attacks per rewarded trials	Two-way ANOVA	Interaction	*F*_(6, 384)_ = 0.9399	0.4662
Figure 1*E*. Attack trials per day	Two-way ANOVA	Interaction	*F*_(6, 384)_ = 0.5290	0.7863
Figure 1*F*. Sniffing location face	Mixed effects with Geisser–Greenhouse correction	Interaction	*F*_(7, 226)_ = 1.739	0.1011
		Day	*F*_(4.233, 270.9)_ = 5.119	0.0004
		Contingency	*F*_(1, 64)_ = 2.759	0.1016
Figure 1*F*. Sniffing location body	Mixed effects with Geisser–Greenhouse correction	Interaction	*F*_(7, 222)_ = 2.578	0.0142
		Day	*F*_(3.236, 102.6)_ = 1.432	0.2358
		Contingency	*F*_(1, 64)_ = 0.6595	0.4197
Figure 1*F*. Sniffing location anogenital	Mixed effects with Geisser–Greenhouse correction	Interaction	*F*_(7, 341)_ = 1.429	0.1926
		Day		
		Contingency		
Figure 1*G*. Attack locations	Two-way ANOVA	Contingency	*F*_(1, 30)_ = 0.02502	0.8754
		Body part	*F*_(2, 30)_ = 330.9	<0.0001
Figure 1*I*. Attack duration by day	Mixed effects with Geisser–Greenhouse correction	Interaction	*F*_(6, 351)_ = 0.8963	0.4976
Figure 1*I*. Attack duration averages	Unpaired *t* test	Contingency	64.0	0.186012
Figure 1*I*. Defensive duration by day	Mixed effects with Geisser–Greenhouse correction	Interaction	*F*_(6, 351)_ = 1.184	0.3143
		Day		
		Contingency		
Figure 1*I*. Defensive duration averages	Unpaired *t* test	Contingency	DF = 64.0	0.232163
Figure 1*I*. Escape duration by day	Mixed effects with Geisser–Greenhouse correction	Interaction	*F*_(6, 351)_ = 2.332	0.0320
		Day	*F*_(3.932, 230.0)_ = 8.992	<0.0001
		Contingency	*F*_(1, 64)_ = 5.043	0.0282
Figure 1*I*. Escape duration averages	Unpaired *t* test	Contingency	DF = 64.0	0.028175
Figure 1*I*. Attack SHAP animal distances	Two-way ANOVA main effects	Time	*F*_(5, 5)_ = 4.821	0.0546
		Contingency	*F*_(1, 5)_ = 0.002795	0.9599
Figure 1*I*. Attack SHAP intruder movement	Two-way ANOVA main effects	Time	*F*_(5, 5)_ = 50.91	0.0003
		Contingency	*F*_(1, 5)_ = 4.275	0.0935
Figure 1*I*. Attack SHAP intruder and resident movement	Two-way ANOVA main effects	Time	*F*_(5, 5)_ = 152.6	<0.0001
		Contingency	*F*_(1, 5)_ = 8.373	0.0340
Figure 1*I*. Attack SHAP resident movement	Two-way ANOVA main effects	Time	*F*_(5, 5)_ = 71.67	0.0001
		Contingency	*F*_(1, 5)_ = 9.371	0.0281
Figure 1*I*. Attack SHAP intruder shape	Two-way ANOVA main effects	Time	*F*_(5, 5)_ = 157.2	<0.0001
		Contingency	*F*_(1, 5)_ = 47.40	0.0010
Figure 1*I*. Attack SHAP intruder and resident shape	Two-way ANOVA main effects	Time	*F*_(5, 5)_ = 131.2	<0.0001
		Contingency	*F*_(1, 5)_ = 10.84	0.0216
Figure 1*I*. Attack SHAP resident shape	Two-way ANOVA main effects	Time	*F*_(5, 5)_ = 57.29	0.0002
		Contingency	*F*_(1, 5)_ = 1.435	0.2847
Figure 1*I*. Attack SHAP intruder shape	Two-way ANOVA main effects	Time	*F*_(5, 5)_ = 12.45	0.0076
		Contingency	*F*_(1, 5)_ = 160.8	<0.0001
Figure 1*I*. Attack SHAP intruder and resident shape	Two-way ANOVA main effects	Time	*F*_(5, 5)_ = 131.2	<0.0001
		Contingency	*F*_(1, 5)_ = 10.84	0.0216
Figure 1*I*. Attack SHAP resident shape	Two-way ANOVA main effects	Time	*F*_(5, 5)_ = 57.29	0.0002
		Contingency	*F*_(1, 5)_ = 1.435	0.2847
Figure 1*I*. Defensive SHAP animal distances	Two-way ANOVA main effects	Time	*F*_(5, 5)_ = 2.548	0.1639
		Contingency	*F*_(1, 5)_ = 59.99	0.0006
Figure 1*I*. Defensive SHAP intruder movement	Two-way ANOVA main effects	Time	*F*_(5, 5)_ = 8.430	0.0177
		Contingency	*F*_(1, 5)_ = 28.48	0.0031
Figure 1*I*. Defensive SHAP intruder and resident movement	Two-way ANOVA main effects	Time	*F*_(5, 5)_ = 5.999	0.0357
		Contingency	*F*_(1, 5)_ = 19.04	0.0073
Figure 1*I*. Defensive SHAP resident movement	Two-way ANOVA main effects	Time	*F*_(5, 5)_ = 8.750	0.0163
		Contingency	*F*_(1, 5)_ = 21.58	0.0056
Figure 1*I*. Attack SHAP intruder shape	Two-way ANOVA main effects	Time	*F*_(5, 5)_ = 12.45	0.0076
		Contingency	*F*_(1, 5)_ = 160.8	<0.0001
Figure 1*I*. Defensive SHAP intruder and resident shape	Two-way ANOVA main effects	Time	*F*_(5, 5)_ = 6.785	0.0278
		Contingency	*F*_(1, 5)_ = 33.59	0.0022
Figure 1*I*. Defensive SHAP resident shape	Two-way ANOVA main effects	Time	*F*_(5, 5)_ = 9.875	0.0126
		Contingency	*F*_(1, 5)_ = 12.92	0.0156
Figure 1*I*. Escape SHAP animal distances	Two-way ANOVA main effects	Time	*F*_(5, 5)_ = 1.508	0.3316
		Contingency	*F*_(1, 5)_ = 20.82	0.0060
Figure 1*I*. Escape SHAP intruder movement	Two-way ANOVA main effects	Time	*F*_(5, 5)_ = 3.747	0.0867
		Contingency	*F*_(1, 5)_ = 14.19	0.0131
Figure 1*I*. Escape SHAP intruder and resident movement	Two-way ANOVA main effects	Time	*F*_(5, 5)_ = 6.232	0.0331
		Contingency	*F*_(1, 5)_ = 7.987	0.0368
Figure 1*I*. Escape SHAP resident movement	Two-way ANOVA main effects	Time	*F*_(5, 5)_ = 5.830	0.0378
		Contingency	*F*_(1, 5)_ = 3.561	0.1178
Figure 1*I*. Escape SHAP intruder shape	Two-way ANOVA main effects	Time	*F*_(5, 5)_ = 1.362	0.3716
		Contingency	*F*_(1, 5)_ = 11.11r	0.0207
Figure 1*I*. Escape SHAP intruder and resident shape	Two-way ANOVA main effects	Time	*F*_(5, 5)_ = 35.13	0.1039
		Contingency	*F*_(1, 5)_ = 0.9565	0.2782
Figure 1*I*. Escape SHAP resident shape	Two-way ANOVA main effects	Time	*F*_(5, 5)_ = 3.735	0.0872
		Contingency	*F*_(1, 5)_ = 11.82	0.0185

### Contingent and noncontingent aggression evoke distinct regional Fos activation patterns across forebrain hypothalamic, striatal, and cortical networks

**Figure 2.**
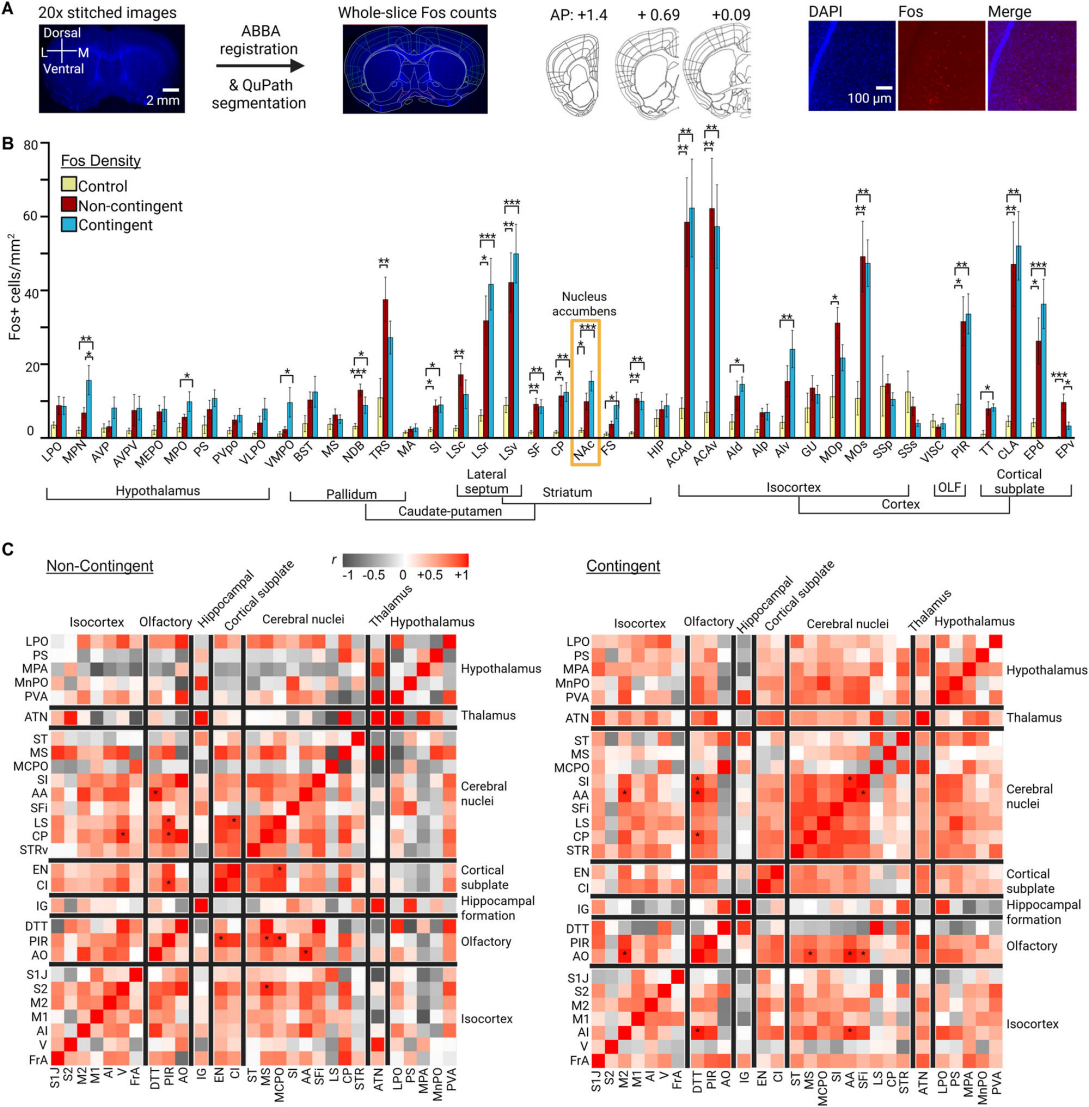
Contingent and noncontingent aggression are characterized by significantly different forebrain neural activity patterns. ***A***, Overview of pipeline used for imaging, stitching, registration to the Allen brain atlas, and automated cell counts using QuPath segmentation. ***B***, Fos-labeled cells (cells/square millimeter) in brain regions across control (*n* = 9), noncontingent (*n* = 8), and contingent (*n* = 9) groups shown. NAc Fos expression differed compared with the control group in noncontingent and contingent mice. Other brain regions that had significant differences between control, noncontingent, and contingent mice include MPN; MPO; VMPO; NDB; TRS; SI; LSc; LSr; LSv; SF; CP; FS; OT; ACAd; ACAv; Ald; Alv; MOp; MOs; PIR; TT; CLA; EPd; and EPv. See Table 2 for full region names and complete statistics, *p < 0.05, **p < 0.01, and ***p < 0.001. ***C***, Regional correlation heatmaps for noncontingent and contingent mice based on mean Fos^+^ density data produced using the SMARTTR pipeline. Asterisks denote significant correlations, *p < 0.05.

To identify brain regions differentially activated by contingent versus noncontingent aggressive behavior, we quantified Fos immunoreactivity across multiple forebrain regions following behavioral testing. Fos immunolabeling across the forebrain was quantified by registering serial 40 µm forebrain sections to the Allen Brain Mouse Reference atlas and detecting Fos counts using QuPath segmentation (Bankhead et al., 2017; Fig. 2*A*). A total of 41 nuclei across 7 gross brain regions were analyzed: hypothalamus, pallidum, lateral septum, striatum, isocortex, olfactory cortex, and cortical subplate. Quantitative analysis of Fos^+^ cell density (cells/square millimeter) in 25 out of the 41 total nuclei demonstrated significant differences in neural activation among control, noncontingent, and contingent groups (Fig. 2*B*; see Table 2 for full statistical analysis; Jin et al., 2025). The control group was behaviorally naive singly housed CD-1 mice. Brains were collected 70–90 min after the start of testing for the test group or directly from the home cage for the control group.

**Table 2.** Detailed statistics for Figure 2*B* data

	Brain region	One-way ANOVA	Tukey's multiple-comparisons test	*p* value
MPN	Medial preoptic nucleus	*F*_(2, 22)_ = 8.3333		0.0020
			Contingent versus noncontingent	0.0458
			Contingent versus no handling control	0.0015
			Noncontingent versus no handling control	0.3499
AVP	Anteroventral preoptic nucleus	*F*_(2, 17)_ = 1.992		0.1670
			Contingent versus noncontingent	0.267
			Contingent versus no handling control	0.2151
			Noncontingent versus no handling control	0.9907
AVPV	Anteroventral periventricular nucleus	*F*_(2, 8)_ = 0.9741		0.4182
			Contingent versus noncontingent	0.9918
			Contingent versus no handling control	0.4167
			Noncontingent versus no handling control	0.549
MEPO	Median preoptic nucleus	*F*_(2, 20)_ = 2.067		0.1528
			Contingent versus noncontingent	0.9734
			Contingent versus no handling control	0.1863
			Noncontingent versus no handling control	0.2437
MPO	Medial preoptic area	*F*_(2, 23)_ = 4.028		0.0316
			Contingent versus noncontingent	0.2546
			Contingent versus no handling control	0.0252
			Noncontingent versus no handling control	0.5171
PS	Parastrial nucleus	*F*_(2, 16)_ = 2.260		0.1367
			Contingent versus noncontingent	0.6311
			Contingent versus no handling control	0.1162
			Noncontingent versus No handling control	0.4856
PVpo	Periventricular hypothalamic nucleus (preoptic part)	*F*_(2, 22)_ = 2.799		0.0826
			Contingent versus noncontingent	0.7827
			Contingent versus no handling control	0.0782
			Noncontingent versus no handling control	0.2665
VLPO	Ventrolateral preoptic nucleus	*F*_(2, 22)_ = 3.182		0.0611
			Contingent versus noncontingent	0.3618
			Contingent versus no handling control	0.0492
			Noncontingent versus no handling control	0.5319
VMPO	Ventromedial preoptic nucleus	*F*_(2, 21)_ = 4.678		0.0209
			Contingent versus noncontingent	0.0613
			Contingent versus no handling control	0.0228
			Noncontingent versus no handling control	0.9079
BST	Bed nuclei of the stria terminalis	*F*_(2, 23)_ = 2.235		0.1297
			Contingent versus noncontingent	0.8753
			Contingent versus no handling control	0.1259
			Noncontingent versus no handling control	0.3172
MS	Medial septal nucleus	*F*_(2, 19)_ = 0.7494		0.4861
			Contingent versus noncontingent	0.8143
			Contingent versus no handling control	0.7634
			Noncontingent versus no handling control	0.456
NDB	Diagonal band nucleus	*F*_(2, 23)_ = 8.998		0.0013
			Contingent versus noncontingent	0.2023
			Contingent versus no handling control	0.049
			Noncontingent versus no handling control	0.0009
TRS	Triangular nucleus of septum	*F*_(2, 23)_ = 6.564		0.0056
			Contingent versus noncontingent	0.3639
			Contingent versus no handling control	0.0831
			Non-contingent versus No handling control	0.0044
MA	Magnocellular nucleus	*F*_(2, 23)_ = 0.6994		0.5071
			Contingent versus noncontingent	0.9465
			Contingent versus no handling control	0.4941
			Noncontingent versus no handling control	0.707
SI	Substantia innominata	*F*_(2, 23)_ = 6.383		0.0062
			Contingent versus noncontingent	0.9903
			Contingent versus no handling control	0.011
			Noncontingent versus no handling control	0.0186
LSc	Lateral septal nucleus, caudal part	*F*_(2, 23)_ = 6.660		0.0052
			Contingent versus noncontingent	0.4021
			Contingent versus no handling control	0.07
			Noncontingent versus no handling control	0.0043
LSr	Lateral septal nucleus, rostral part	*F*_(2, 23)_ = 11.16		0.0004
			Contingent versus noncontingent	0.4463
			Contingent versus no handling control	0.0004
			Noncontingent versus no handling control	0.0103
LSv	Lateral septal nucleus, ventral part	*F*_(2, 22)_ = 10.26		0.0007
			Contingent versus noncontingent	0.6952
			Contingent versus no handling control	0.0008
			Noncontingent versus no handling control	0.007
SF	Septofimbrial nucleus	*F*_(2, 21)_ = 9.203		0.0013
			Contingent versus noncontingent	0.9397
			Contingent versus no handling control	0.0053
			Noncontingent versus no handling control	0.0024
CP	Caudoputamen	*F*_(2, 23)_ = 7.951		0.0024
			Contingent versus noncontingent	0.9441
			Contingent versus no handling control	0.0038
			Noncontingent versus no handling control	0.0107
NAc	Nucleus accumbens	*F*_(2, 21)_ = 10.69		0.0006
			Contingent versus noncontingent	0.1598
			Contingent versus no handling control	0.0004
			Noncontingent versus no handling control	0.0353
FS	Fundus of striatum	*F*_(2, 22)_ = 3.514		0.0474
			Contingent versus noncontingent	0.6342
			Contingent versus no handling control	0.0412
			Noncontingent versus no handling control	0.243
OT	Olfactory tubercle	*F*_(2, 23)_ = 11.07		0.0004
			Contingent versus noncontingent	0.9423
			Contingent versus no handling control	0.0018
			Noncontingent versus no handling control	0.0011
HIP	Hippocampal region	*F*_(2, 23)_ = 0.4808		0.6243
			Contingent versus noncontingent	0.9605
			Contingent versus no handling control	0.6116
			Noncontingent versus no handling control	0.7906
ACAd	Anterior cingulate area, dorsal part	*F*_(2, 23)_ = 8.773		0.0015
			Contingent versus noncontingent	0.9639
			Contingent versus no handling control	0.0027
			Noncontingent versus no handling control	0.0065
ACAv	Anterior cingulate area, ventral part	*F*_(2, 23)_ = 9.383		0.0010
			Contingent versus noncontingent	0.9381
			Contingent versus no handling control	0.004
			Noncontingent versus no handling control	0.0023
AId	Agranular insular area, dorsal part	*F*_(2, 21)_ = 3.517		0.0482
			Contingent versus non-contingent	0.7077
			Contingent versus no handling control	0.0434
			Noncontingent versus no handling control	0.197
Alp	Agranular insular area, posterior part	*F*_(2, 23)_ = 3.164		0.0611
			Contingent versus noncontingent	0.9994
			Contingent versus no handling control	0.0896
			Noncontingent versus no handling control	0.1085
Alv	Agranular insular area, ventral part	*F*_(2, 21)_ = 6.435		0.0066
			Contingent versus noncontingent	0.2805
			Contingent versus no handling control	0.0048
			Noncontingent versus no handling control	0.1347
GU	Gustatory area	*F*_(2, 23)_ = 0.6956		0.5090
			Contingent versus noncontingent	0.9288
			Contingent versus no handling control	0.7052
			Noncontingent versus no handling control	0.4969
MOp	Primary motor area	*F*_(2, 23)_ = 4.573		0.0213
			Contingent versus noncontingent	0.3428
			Contingent versus no handling control	0.2508
			Noncontingent versus no handling control	0.0162
MOs	Secondary motor area	*F*_(2, 23)_ = 10.08		0.0007
			Contingent versus noncontingent	0.9813
			Contingent versus no handling control	0.0024
			Noncontingent versus no handling control	0.002
SSp	Primary somatosensory area	*F*_(2, 23)_ = 0.1970		0.8225
			Contingent versus noncontingent	0.8348
			Contingent versus no handling control	0.8722
			Noncontingent versus no handling control	0.9956
SSs	Supplemental somatosensory area	*F*_(2, 23)_ = 1.416		0.2630
			Contingent versus noncontingent	0.6662
			Contingent versus no handling control	0.2333
			Noncontingent versus no handling control	0.7276
VISC	Visceral area	*F*_(2, 23)_ = 0.3471		0.7104
			Contingent versus noncontingent	0.8898
			Contingent versus no handling control	0.9226
			Noncontingent versus no handling control	0.6868
PIR	Piriform area	*F*_(2, 23)_ = 7.371		0.0034
			Contingent versus noncontingent	0.9573
			Contingent versus no handling control	0.0055
			Noncontingent versus no handling control	0.0135
TT	Taenia tecta	*F*_(2, 7)_ = 5.382		0.0384
			Contingent versus noncontingent	0.9909
			Contingent versus no handling control	0.0436
			Noncontingent versus no handling control	0.0503
CLA	Claustrum	*F*_(2, 23)_ = 10.21		0.0007
			Contingent versus noncontingent	0.9069
			Contingent versus no handling control	0.0011
			Noncontingent versus no handling control	0.0042
Epd	Endopiriform nucleus, dorsal part	*F*_(2, 23)_ = 10.17		0.0007
			Contingent versus noncontingent	0.3905
			Contingent versus no handling control	0.0006
			Noncontingent versus no handling control	0.0191
Epv	Endopiriform nucleus, ventral part	*F*_(2, 18)_ = 10.32		0.0010
			Contingent versus noncontingent	0.0258
			Contingent versus no handling control	0.3977
			Noncontingent versus no handling control	0.0009

#### Hypothalamus

Within the hypothalamus, Fos density was significantly increased in the medial preoptic nucleus (MPN; *F*_(2, 22)_ = 8.3333, *p* ≤ 0.0020) and medial preoptic area (MPO; *F*_(2, 23)_ = 4.028; *p* = 0.0316) compared with controls. Both contingent and noncontingent groups exhibited elevated activation in these regions, with the contingent condition showing the greatest increase. Additional hypothalamic nuclei, including the lateral preoptic area (LPO), anteroventral periventricular nucleus (AVPV), median preoptic nucleus (MEPO), and ventromedial preoptic area (VMPO), displayed modest but nonsignificant trends toward increased Fos expression.

#### Pallidum and septal regions

In pallidal structures, significant group effects emerged in the diagonal band nucleus (NDB; *F*_(2, 23)_ = 8.998; *p* = 0.0013) and triangular septal nucleus (TRS; *F*_(2, 23)_ = 6.564; *p* = 0.0056). Both NDB and TRS showed elevated Fos labeling in noncontingent relative to control mice, with contingent animals exhibiting comparable but slightly reduced responses. The substantia innominata (SI) also displayed increased Fos activation (*F*_(2, 23)_ = 6.383; *p* = 0.0062).

#### Lateral septum

Fos activation was robust across all divisions of the lateral septum, with significant effects in the caudal (LSc; *p* = 0.0052; *F*_(2, 23)_ = 6.660), rostral (LSr; *p* = 0.0004; *F*_(2, 23)_ = 11.16), and ventral (LSv; *p* = 0.0007; *F*_(2, 22)_ = 10.26) subdivisions. These regions showed strong recruitment in both aggression groups relative to controls, with noncontingent aggression yielding slightly greater Fos density than contingent aggression.

#### Striatum

Within striatal territories, significant activation was detected in the caudate putamen (CP; *F*_(2, 23)_ = 7.951; *p* = 0.0024), fundus of striatum (FS; *F*_(2, 22)_ = 3.514; *p* ≤ 0.0474), and NAc (*F*_(2, 21)_ = 10.69; *p* = 0.0006). Post hoc tests confirmed that both contingent and noncontingent groups exhibited elevated Fos expression in the NAc relative to controls, with no significant difference between aggression conditions. The olfactory tubercle (OT) also displayed increased activation in both groups (*p* < 0.01), consistent with its involvement in motivational and sensory integration circuits.

#### Isocortex and cortical subplate

Widespread cortical activation was observed in regions of the isocortex and subplate. Both anterior cingulate subdivisions (ACAd, ACAv) and the agranular insular cortex (AId, AIv) showed significant main effects of group (*p* = 0.0015, 0.0010, 0.0482, 0.0066, respectively), with Fos levels elevated in both aggression groups relative to controls. Motor cortical regions (MOp, MOs) exhibited moderate increases (*p* = 0.0213, 0.0007 respectively), while sensory areas (SSp, SSs, VISC) showed smaller, nonsignificant effects. Within subplate structures, strong activation was evident in the claustrum (CLA; *p* = 0.0007) and dorsal and ventral endopiriform nuclei (EPd, EPv; *p* = 0.0007, 0.0010, respectively), particularly in noncontingent animals. The piriform cortex (PIR) and taenia tecta (TT) also demonstrated elevated Fos density (*p* = 0.0034, 0.0384, respectively), with contingent mice showing relatively higher values.

Collectively, these data reveal that contingent and noncontingent aggression engage overlapping but topographically distinct networks of neural activation. Noncontingent aggression elicited widespread Fos induction across septal, striatal, and subplate regions, while contingent aggression was associated with stronger recruitment of hypothalamic and cortical regions, suggesting differential circuit mechanisms underlying motivated versus defensive aggression.

Correlation analyses of regional Fos activity further revealed that contingent and noncontingent aggression were characterized by distinct network-wide activity profiles (Fig. 2*C*). Contingent aggression produced a more cohesive and positively correlated pattern of activation across cortical and subcortical structures, whereas noncontingent aggression exhibited weaker or more regionally restricted correlations. Together, these data suggest that appetitive and reactive aggression recruit overlapping but distinct ensembles of limbic and cortical circuits involved in motivational and sensorimotor processing.

### Reduced NAc NLGN2 expression is associated with heightened aggression in both contingent and noncontingent aggression self-administration

Since we observed similar upregulation of NAc Fos expression in both contingent and noncontingent aggression groups, we aimed to further investigate molecular substrates within NAc that could differentiate noncontingent and contingent self-administration. As a targeted hypothesis, based on our prior published findings (Heshmati et al., 2018; Golden et al., 2019), we examined NLGN-2 levels in the NAc. NLGN-2 is a postsynaptic inhibitory protein that is shown to regulate aggression and stress behavior (van der Kooij et al., 2014; Kohl et al., 2015). We previously published that NLGN-2 regulates aggression and dominance behavior in mice (Heshmati et al., 2018). Prior studies also show the role of NLGN-2 in modulating aggression in the hippocampus and prefrontal cortex (Kohl et al., 2013, 2015; van der Kooij et al., 2014).

**Figure 3.**
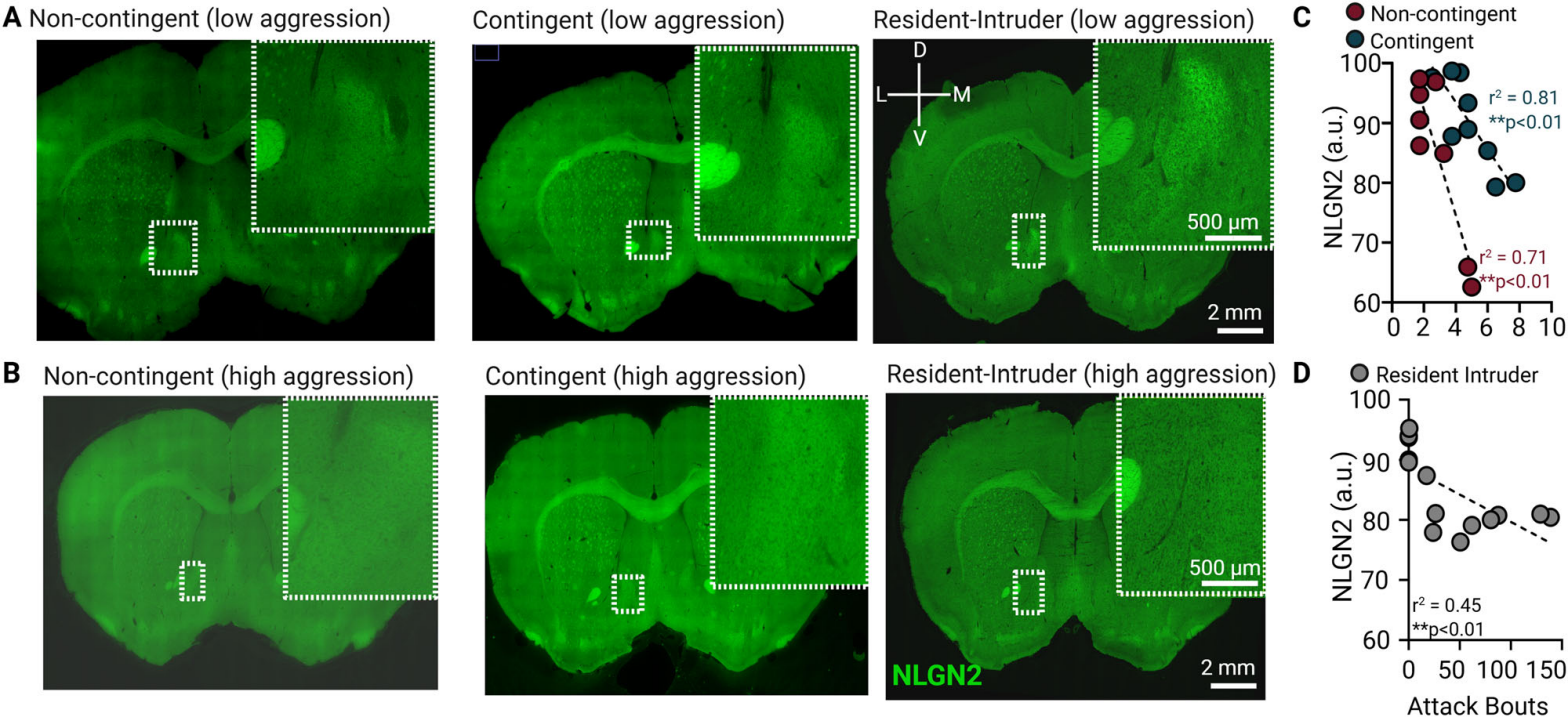
Reduced NAc NLGN2 expression correlates with increased aggression in CD-1 mice, regardless of operant contingency. Representative images of NLGN2 protein expression in NAc from (***A***) low aggression CD-1 mice (<5 attack bouts) and (***B***) high aggression mice. ***C***, Reduced NLGN2 mean fluorescence correlates with higher number of attack bouts in both noncontingent (*r*^2^ = 0.71; **p < 0.01) and contingent mice (*r*^2^ = 0.81; **p < 0.01) during 2 min operant trials. ***D***, Reduced NLGN2 mean fluorescence correlates with the higher number of attack bouts in 10 min resident–intruder testing (*r*^2^ = 0.45; **p < 0.01). See Table 3 for full statistics.

To investigate whether synaptic inhibitory signaling within the NAc is related to individual differences in aggression, we examined expression of NAc NLGN2 in CD-1 mice following contingent (appetitive) and noncontingent (reactive) aggression self-administration testing as well as resident–intruder (reactive) testing in their home cage (Fig. 3). Regardless of contingency, we found that low aggression mice had higher expression of NLGN2 (Fig. 3*A*) than mice with high aggression (Fig. 3*B*), as defined by higher number of attack bouts. Regression analyses revealed a strong inverse relationship between NLGN2 signal intensity and the number of attack bouts in both noncontingent (*r^2^* = 0.7104; *p* = 0.0043) and contingent (*r^2^* = 0.8088; *p* = 0.0024) groups (Fig. 3*C*).

To confirm our findings that NAc NLGN-2 correlates with aggression severity outside the contingency context, we also quantified NLGN-2 immunolabeling in traditional resident–intruder tested mice. Reduced NLGN-2 expression again correlated with increased aggression in resident–intruder testing (Fig. 3*D*; *r*^2^ = 0.4479; *p* = 0.0089; see Table 3 for full statistics). Together, these data indicate that reduced NLGN2 expression within the NAc is a consistent molecular substrate of heightened aggression regardless of contingency.

**Table 3.** Detailed statistics for Figure 3

Figure	Panel	Test		*p* value
3	*C*	Simple linear regression	*Y* = −3.956*X + 109.3; *R* squared = 0.7104	0.0043
		Simple linear regression	*Y* = −8.858*X + 110.0; *R* squared = 0.8088	0.0024
	*D*	Simple linear regression	*Y* = −0.2271*X + 71.91; *R* squared = 0.4479	0.0089

## Discussion

Aggressive behavioral phenotypes in humans include reactive and appetitive aggression (Moran et al., 2014; de Almeida et al., 2015; Chester and DeWall, 2016). While aggression can be adaptive, working to secure safety, food, and mates, aggression can also be maladaptive, manifesting in violence that negatively impacts quality of life. Pathological aggression is thought to arise from the overactivation of evolutionarily conserved reward pathways, resembling mechanisms underlying drug addiction (Golden and Shaham, 2018). Despite the substantial burden of maladaptive aggression for individuals and caregivers, few effective treatments for maladaptive aggression exist. Shedding light on the mechanisms that differentiate aggression phenotypes is essential for developing targeted interventions.

To determine whether neural activity patterns differ between contingent (appetitive) and noncontingent (reactive) aggression in males, we examined Fos expression following operant-reinforced aggression testing under noncontingent and contingent contexts. Female mice were excluded due to their relative lack of attack behavior during contingent social self-administration (Aubry et al., 2022). Fos immunolabeling in forebrain slices revealed significant activation across multiple cortical, striatal, and hypothalamic regions in both contingent and noncontingent groups compared with controls, indicating widespread recruitment of aggression-related circuits. However, few regions showed significant differences between contingent and noncontingent mice. The NAc showed significantly elevated activity in both aggression phenotypes, aligning with prior findings that NAc activity increases following aggressive encounters (Golden et al., 2019). The ventral endopiriform nucleus (EPv) was one of the few regions differing between groups, suggesting it may represent a previously underappreciated node in aggression-related processing. Given the limited literature on the EPv, its potential role in aggression and its connectivity with the amygdala warrant further investigation.

Beyond absolute activity levels, we also examined Fos coactivation networks using the open-source SMARTTR workflow (Jin et al., 2025) to understand whether contingent and noncontingent aggression recruit broader patterns of coordinated activity. Cross-correlation analyses of regional Fos density demonstrated that contingent aggression produced more cohesive and positively correlated activity across cortical and subcortical structures, whereas noncontingent aggression was characterized by weaker and more regionally restricted correlations. These findings suggest that contingent aggression engages more integrated motivational and motor networks, while noncontingent aggression involves more localized responses. Overall, these network activity patterns suggest distinct patterns of forebrain activity underlie differences in aggression motivation.

We specifically examined the NAc due to its known role in modulating aggression, previously observed after chemogenetic modulation of medium spiny neurons after NAc NLGN-2 manipulation (Heshmati et al., 2018, 2020; Golden et al., 2019). Neuroligin mutations are associated with neuropsychiatric disorders such as autism and schizophrenia (Jamain et al., 2003; Südhof, 2008; Heshmati et al., 2018, 2020) and a possible underlying factor in anxiety and depression (Parente et al., 2017). Consistent with preclinical observations, NLGN-2 within the NAc is also downregulated in the postmortem tissue of patients with major depressive disorder (Heshmati et al., 2020). We found that reduced NAc NLGN-2 expression was associated with heightened aggression, regardless of whether behavior was appetitively or reactively motivated. This inverse relationship was replicated in resident–intruder testing, suggesting decreased inhibitory synaptic signaling in NAc may be a common molecular correlate of higher aggression independent of behavioral context. Although contingency did not significantly alter overall NLGN-2 levels, our findings suggest that NLGN-2 contributes to aggression severity and may represent a shared mechanism underlying contingent and noncontingent aggression. However, one limitation of this study is that we do not directly investigate a causal role or identify cell type and circuit specificity for NAc NLGN2, as well as the time course of expression. These will be the focus of future studies.

A key observation is that both aggression types elevate NAc Fos, though reduced NLGN2 correlates with greater aggression. Given that NLGN2 dysfunction is implicated in autism spectrum disorder, schizophrenia, and mood disorders, our findings suggest shared inhibitory mechanisms may underlie aggression across diagnostic boundaries. Identifying how NLGN2-dependent synaptic regulation in NAc shapes aggression circuits may help refine therapeutic strategies targeting maladaptive aggression without broadly suppressing motivation.

Behaviorally, while SHAP-based explainable machine learning identified significant differences in the features defining aggression bouts, traditional ethological measures such as bout number, duration, and attack target location did not distinguish between aggression phenotypes. This suggests that contingent and noncontingent aggression manifest as behaviorally robust, superficially similar repertoires despite distinct motivational and molecular underpinnings. These findings underscore the importance of explainable computational approaches for distinguishing motivation-dependent states that are behaviorally indistinguishable at the surface level. By pairing explainability metrics with ethological validation, we can bridge traditional behavioral analysis with data-driven insight into internal motivational states.

Together, these data indicate that behavioral repertoires of aggression are conserved across motivational contexts, but the underlying neural networks and molecular markers might diverge. Contingent and noncontingent aggression produce overlapping yet distinct Fos activation profiles, with contingent aggression recruiting more cohesive cortical–subcortical networks. In contrast, NAc NLGN-2 expression inversely correlates with aggression intensity across both conditions, implicating inhibitory synaptic mechanisms in modulating aggression regardless of its motivational underpinning. We suggest that molecularly dissociable features do not necessarily produce observable differences in attack behavior and highlight the importance of integrating explainable computational, molecular, and circuit-level analyses to distinguish the neural bases of contingent and noncontingent aggression.

## Synthesis

Reviewing Editor: Charlotte Cornil, Universite de Liege Decisions are customarily a result of the Reviewing Editor and the peer reviewers coming together and discussing their recommendations until a consensus is reached. When revisions are invited, a fact-based synthesis statement explaining their decision and outlining what is needed to prepare a revision will be listed below. The following reviewer(s) agreed to reveal their identity: Brian Trainor.

Your manuscript has now been reviewed by two experts who both agree that the question is interesting and relevant and that for the most part the manuscript is technically rigorous, clearly written and methodologically thorough. However, some aspects of the methods/results need clarification or may benefit from further discussion. They also share the feeling that the data could have been exploited more and that further analyses could bring more novelty and increase the impact of this work.

Methodological clarifications

- Information regarding an animal license or ethical committee approval should be provided in the methods section.

- It should be clearly stated in the abstract and methods why only male mice were used.

- The large-scale Fos immunoreactivity analysis across 41 forebrain nuclei is impressive and provides a broad systems-level video of aggression-related activity. It wasn't clear whether the authors did any FDR correction. If not, it would be good for the authors to address this in the revision.

- Was both core and shell included in this analysis?

- The authors provide a nice definition for appetitive aggression and how it is measured in the introduction, the same should be done for reactive aggression. How can the authors be sure that non-contingent aggression is reactive? Interpretation might be simpler to stick with contingent and non-contingent since those conditions are easier to define. It would also be useful for the authors to clarifying how conserved behavioral repertories inform the neural analyses.

- Along the same line, how does the neuronal activity presented after lever pressing to have access to an intruder compare to the Fos elicited by the resident intruder test? Comparing Fos expression in both contingent and non-contingent groups perfused after displaying the resident intruder test gives somewhat relevant information about the neuronal networks involved in reactive vs proactive aggression. However, this experimental design may not completely address the strongest component of an operant task, which is related to the aggression-seeking behavior (lever pressing). This might be the reason why the authors do not find a difference between the contingent and non-contingent tasks when it comes to network activity. Could the authors clarify this experiment? And give more information about controls regarding the Fos experiment? Alternatively, given that the main hypothesis is that there is a difference between contingent and non-contingent aggression, it seems that the direct comparison between those two groups is more important.

- The photomicrographs of NLGN-2 seemed to have a lot of background. In Fig 3A, the non-contingent and contingent images look like background. The low aggression panel is the only one that has what looks like positive staining to me. It would be beneficial for the authors to include more information about how this antibody has been tested for specificity. Were there any differences in NLGN-2 in the EPv? That seems like a logical place to look.

Expected improvements/clarifications of results:

- The results section would profit from having higher magnifications of representative pictures for immunohistochemistry regarding Fos and NLGN expression, as in the current pictures, one can hardly see differences in expression.

- Regarding the correlation analysis, for Fos and aggression levels presented in Figure 3. Which components of aggressive behavior have been correlated to neuronal activity? Time spent on aggression, number of attacks, time spent attacking? How does previous experience (high baseline aggression first RI ) reflect on those correlations?

Suggested additional analyses

- The authors pre-selected residents for the operant task based on a 3-day assessment of aggression levels using the resident intruder test. That is quite interesting. Did the authors investigate whether baseline (first day of resident intruder test, or average over 3 tests) influences the appetitive and reactive aggression of the animals? Or the learning curve and/or reward-seeking behavior? Additionally, how those baseline levels of aggression reflect on neuronal activity and on Neuroligin expression in the nucleus accumbens. Could the authors provide those analyses and/or speculate on the influence of the animals' baseline aggressive behavior on their operant behavior?

- How NLGN-2 expression correlate with behaviors expressed i) during operant task such as the number of lever presses or number of rewards? And ii) to the animal's previous experience (first RI baseline aggression)? Performing those analyses would help the authors to draw a point about the appetitive vs reactive components of aggressive behavior and NLGN-2. Attack bouts in both groups (contingent and non-contingent) reflect mostly the consummatory and reactive aspects of aggression

Suggested additional points of discussion

- The authors measure Fos expression after the animals were exposed to a resident intruder test. In this context, the animals displayed mostly reactive aggressive behavior towards an intruder released in their home cage. One may wonder whether having the animals either display the contingent or the non-contingent task before perfusion would reflect on the neuronal networks recruited by Fos. Could the authors speculate on that?

- Reactive and proactive aggression have been characterized as two different helms of aggressive behavior, particularly regarding clinical relevance. How do the authors interpret the lack of differences in molecular, neuronal, and behavioral levels for those two types of aggression? Is that related to using animal models? Is the line between reactive and proactive aggression more like a gray zone, instead of a black-and-white separation? What can we learn from it?

Minor comments:

- Be consistent with abbreviation for nucleus accumbens (pick NAc or Acb) across test and figure.

- In the discussion, the authors write: " This finding suggests that NAc activation may represent a common motivational endpoint, while inhibitory synaptic regulation through NLGN2 fine-tunes aggression intensity. This supports a model in which NAc excitation encodes aggression drive, whereas inhibitory balance modulates behavioral expression." Reduced expression and correlation do not necessarily correlate with function, meaning that the authors cannot directly link the expression of NLGN2 with decreased inhibitory tonus in the NAc (further experiments would be necessary to raise this point). Therefore, removing this sentence is strongly suggested.

- The authors mention the influence of NLGN-2 in several psychiatric disorders known to have aggression as a core symptom, such as major depression, schizophrenia, and autism spectrum disorder. However, those disorders are mostly characterized by high levels of reactive aggression. Is NLGN-2 also involved in disorders known to be associated with increased proactive aggression, such as antisocial and conduct disorder? The manuscript would profit from having this information included.
